# Reversing valproic acid-induced autism-like behaviors through a combination of low-frequency repeated transcranial magnetic stimulation and superparamagnetic iron oxide nanoparticles

**DOI:** 10.1038/s41598-024-58871-5

**Published:** 2024-04-06

**Authors:** Masoud Afshari, Shahriar Gharibzadeh, Hamidreza Pouretemad, Mehrdad Roghani

**Affiliations:** 1 Department of Cognitive Psychology, Institute for Cognitive and Brain Sciences, Shahid Beheshti University, Tehran, Iran; 2 Neurophysiology Research Center, Shahed University, Tehran, Iran

**Keywords:** Transcranial magnetic stimulation, Autism spectrum disorder, Valproic acid, Superparamagnetic iron oxide nanoparticles, Nanomedicine, Autism spectrum disorders, Neurotrophic factors

## Abstract

Transcranial magnetic stimulation (TMS) is a neurostimulation device used to modulate brain cortex activity. Our objective was to enhance the therapeutic effectiveness of low-frequency repeated TMS (LF-rTMS) in a rat model of autism spectrum disorder (ASD) induced by prenatal valproic acid (VPA) exposure through the injection of superparamagnetic iron oxide nanoparticles (SPIONs). For the induction of ASD, we administered prenatal VPA (600 mg/kg, I.P.) on the 12.5th day of pregnancy. At postnatal day 30, SPIONs were injected directly into the lateral ventricle of the brain. Subsequently, LF-rTMS treatment was applied for 14 consecutive days. Following the treatment period, behavioral analyses were conducted. At postnatal day 60, brain tissue was extracted, and both biochemical and histological analyses were performed. Our data revealed that prenatal VPA exposure led to behavioral alterations, including changes in social interactions, increased anxiety, and repetitive behavior, along with dysfunction in stress coping strategies. Additionally, we observed reduced levels of SYN, MAP2, and BDNF. These changes were accompanied by a decrease in dendritic spine density in the hippocampal CA1 area. However, LF-rTMS treatment combined with SPIONs successfully reversed these dysfunctions at the behavioral, biochemical, and histological levels, introducing a successful approach for the treatment of ASD.

## Introduction

Autism spectrum disorder (ASD) is a complex condition characterized by repetitive behaviors and difficulties with social interactions, affecting more than 1% of the child population^1^. While genetics play a significant role in its etiology, a growing number of cases are believed to be caused by environmental factors such as maternal separation and exposure to drugs such as valproic acid (VPA)^2–4^.

Evidence suggests that the hippocampus plays a crucial role in the pathophysiology of autism and social interactions^5^. Dendrites and spines are critical structures in neurons for receiving input and integrating signals from other neurons and glial cells. The morphology and density of these structures play a key role in signal integration in brain regions such as the hippocampus^6,7^. Abnormalities in dendrites and synaptic transmission have been increasingly observed in autism patients and animal models^8,9^. Notably, microtubule-associated protein 2 (MAP2) is a protein enriched in dendrites and serves as a marker for synaptic plasticity^10^. On the other hand, synaptic proteins, including synaptophysin (SYN), are involved in synaptic plasticity and transmission^11^. Any changes in the levels of these proteins may be associated with abnormal dendritic and synaptic function. Overall, the health of dendrites and efficient synaptic transmission are essential for normal brain function in different regions, such as the hippocampus.

Physical therapy, including repetitive transcranial magnetic stimulation (rTMS), has become increasingly popular for the prevention and treatment of various medical conditions^12,13^. rTMS involves the application of multiple magnetic pulses to specific areas of the brain cortex to modify its activity^14^. Despite its safety and tolerability, more research is needed before rTMS can be widely recommended as a treatment option for ASD patients^15,16^. There are two types of rTMS treatment: low-frequency rTMS (LF-rTMS) and high-frequency rTMS (HF-rTMS). LF-rTMS, with frequencies ≤ 1 Hz, has inhibitory effects, while HF-rTMS, with frequencies ≥ 5 Hz, has excitatory effects^17^. A study in a maternal separation model of autism revealed that LF-rTMS could modulate synaptic and neuronal activity^18^. Additionally, studies have reported that transcranial magnetic stimulation (TMS) can increase brain-derived neurotrophic factor (BDNF) levels in serum^19^. It has also been noted that BDNF plays a significant role in dendritic growth and spine maturation^20,21^. Another study showed that LF-rTMS improved social behavior, but HF-rTMS did not, due to overexcitation of the brain in a genetic model of ASD^22^. However, TMS has limitations, including limited penetration power and accuracy^23^. Research suggests that deeper parts of the brain, including the hippocampus, play a crucial role in the pathophysiology of ASD^24^. Therefore, developing a new technique for using TMS to stimulate deeper parts of the brain is crucial.

Magnetic nanoparticles such as superparamagnetic iron-oxide nanoparticles (SPIONs) can influence brain activity through magneto-thermal and magnetoelectric mechanisms when exposed to magnetic fields or radiofrequency waves^25–27^. Studies have demonstrated that magnetic stimulation using SPIONs can enhance brain stimulation and improve depression and stroke recovery^28,29^.

Building on this knowledge, it is hypothesized that combining TMS to target both superficial and deeper brain areas with SPIONs, such as the hippocampus, may be more effective in reducing symptoms of ASD.

## Results

### SPIONs characteristics

**Figure 1:**
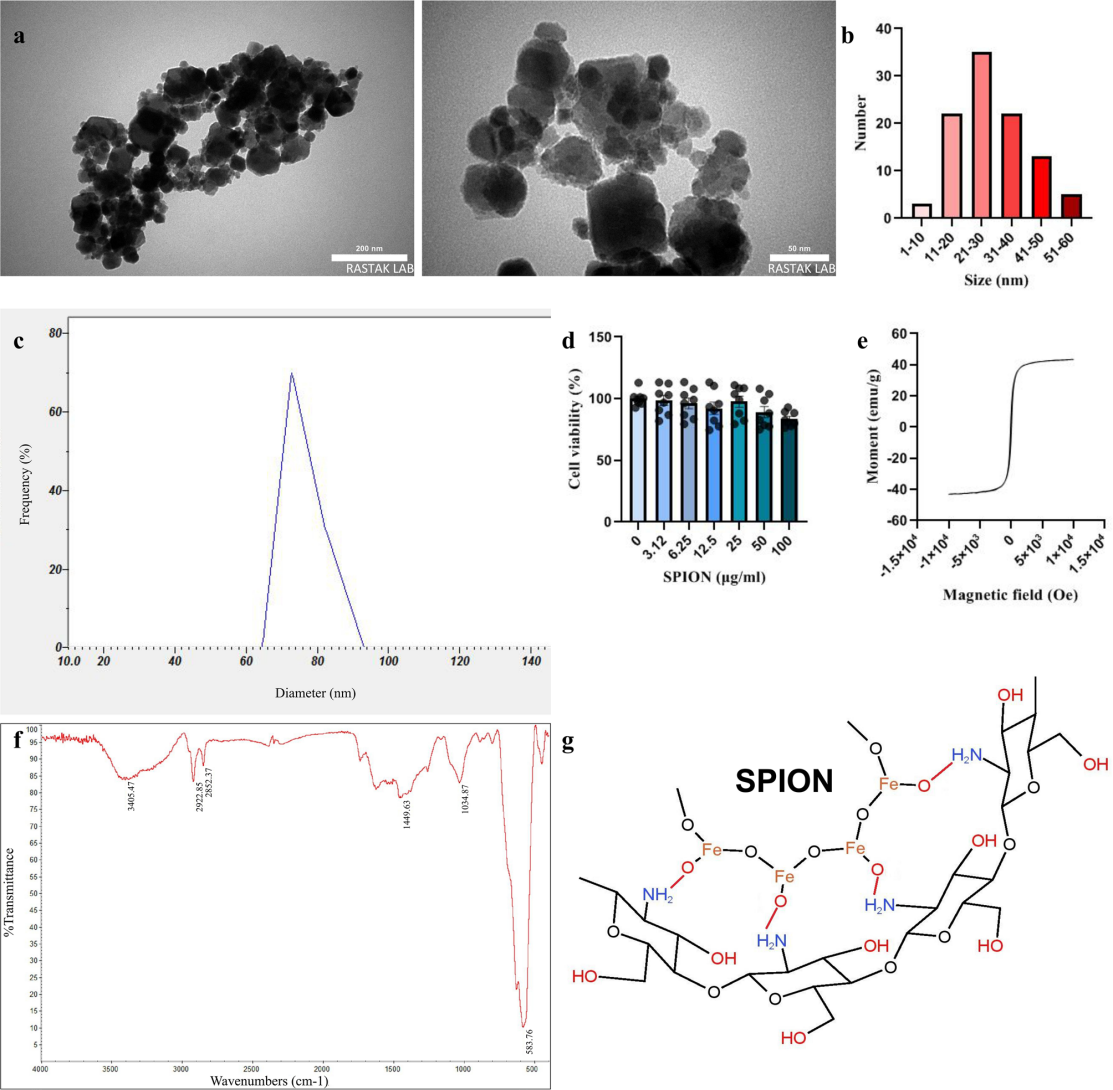
Characteristics of chitosan-coated superparamagnetic iron oxide (Fe_3_O_4_) nanoparticles (SPIONs). (**a**) Transmission electron microscopy (TEM) shows that these particles have a spherical shape, with chitosan well coated around the iron core. (**b**) The mean diameter of these particles was calculated based on the measurement of 100 random particles in TEM images (28.6 nm). (**c**) Dynamic light scattering (DLS) testing revealed the mean hydrodynamic diameter of these particles (71.2 nm). (**d**) The MTT assay of SPION showed no significant toxicity at different concentrations. (**e**) Vibrating sample magnetometer (VSM) provided information on the magnetic hysteresis of chitosan-coated SPIONs. (**f**) Fourier transform infrared spectroscopy (FTIR) analysis showed several distinct bands indicating the presence of iron and chitosan in the final product. These bands include 3445 cm^−1^ (O–H and N–H), 2922 cm^−1^ and 2852 cm^−1^ (C–H), 1449 cm^−1^ (C–N), 1034 cm^−1^ (C–O–C), and 583 cm^−1^ (Fe–O). (**g**) The chemical structure of chitosan-coated SPION (KingDraw).

According to TEM, the nanoparticles displayed an appropriate shape (Fig. 1a) and had a diameter of 28.6 nm after the coating process, calculated using the ImageJ program based on 100 random particles (Fig. 1b). The DLS test revealed that the SPIONs possessed a negative charge (− 11.7 mV) and exhibited a hydrodynamic diameter of 71.2 nm (Fig. 1c).

Furthermore, the MTT assay data demonstrated no significant change in cell viability across all concentrations (0–100 µg/ml) compared to the control group (Fig. 1d). The graph (Fig. 1e) illustrates that the coated nanoparticles exhibit superparamagnetic properties (zero remanence and coercivity) with magnetization saturation at 43 emu/g. Moreover, the FTIR results confirmed the successful binding of Fe_3_O_4_ to chitosan (Fig. 1f). The total SPION content in the hippocampus after 30 days from lateral ventricle injection was 21.45 ± 0.69 µg/ml, whereas it was 9.96 ± 0.14 µg/ml in those that did not receive SPIONs. Figure 1g displays the structure of the coated SPIONs.

### Three-chamber test

**Figure 2:**
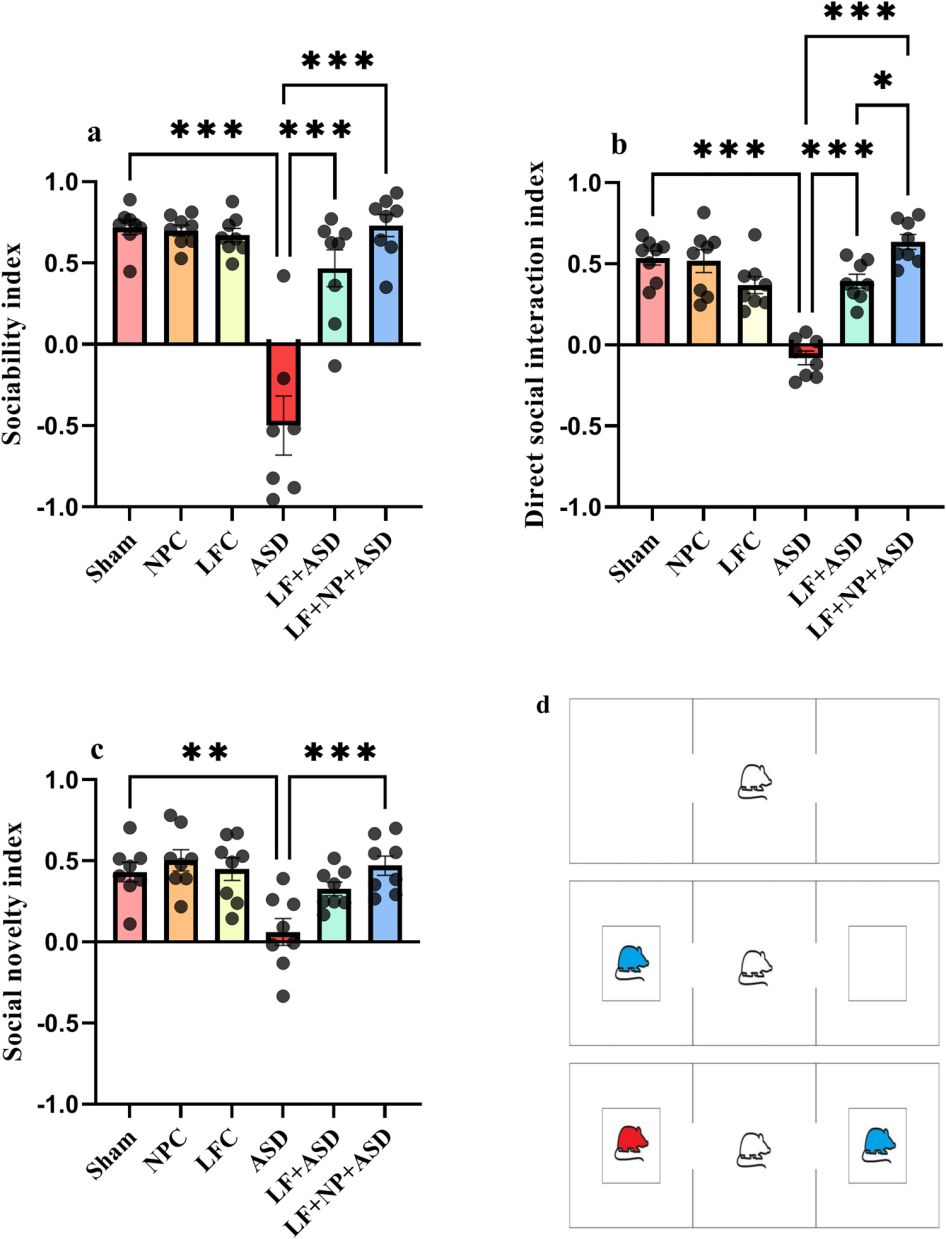
The effect of low-frequency repeated transcranial magnetic stimulation (LF-rTMS) and superparamagnetic iron oxide nanoparticles (SPIONs) on social behavior. (**a**) Sociability index. (**b**) Direct social interaction index. (**c**) Social novelty index. (**d**) This figure illustrating the different stages of the test, including habituation, sociability, and social novelty. (N = 8, mean ± SEM). *p < 0.05, **p < 0.01, ***p < 0.001.

This study, which investigated the effects of LF-rTMS on social behavior, is presented in Fig. 2. The sociability index was calculated by dividing the time spent exploring the social chamber by the total time spent exploring both the social and nonsocial chambers. One-way ANOVA revealed significant main effects of autism and rTMS treatment on the sociability index (F (5, 41) = 25.89, *p* < 0.001; Fig. 2a). The Tukey post hoc test indicated a significant decrease in the sociability index for the ASD group compared to the sham group (*p* < 0.001). Additionally, rTMS treatment significantly increased the sociability index in both the LF + ASD and LF + NP + ASD groups (*p* < 0.001).

The direct social interaction index was defined as the time rats spent sniffing social stimuli minus the time spent sniffing nonsocial stimuli, divided by the sum of these times. Data analysis revealed significant differences among the groups (F (5, 42) = 24.57, *p* < 0.001; Fig. 2b). Post hoc test analysis demonstrated significant distinctions between the ASD group and the sham group (*p* < 0.001). Furthermore, rTMS treatment in both the LF + ASD and LF + NP + ASD groups showed that rats displayed significant social responses compared to untreated autistic rats (*p* < 0.001). In the LF + NP + ASD group, a significant increase in direct social interaction was observed compared to the LF + ASD group (*p* < 0.05).

The social novelty index was defined as the time spent investigating novel social stimuli minus the time spent investigating familiar stimuli, divided by the sum of the abovementioned times. The data revealed significant differences between the experimental groups (F (5, 42) = 6.494, *p* < 0.001; Fig. 2c). Autistic rats exhibited a significant reduction in social novelty preference (*p* < 0.01). The combination of LF-rTMS treatment with SPIONs demonstrated a significant treatment effect (*p* < 0.001). The three-chamber test is presented in Fig. 2d.

### Marble burying test

**Figure 3:**
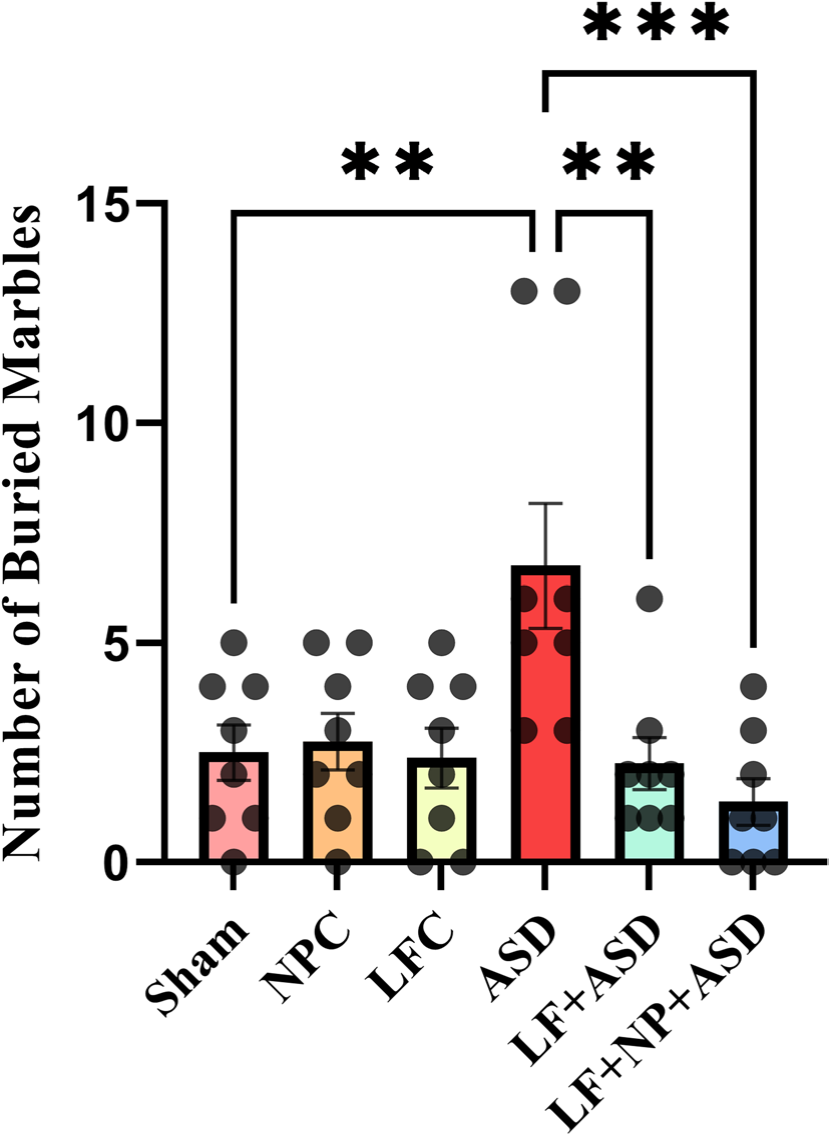
The effect of low-frequency repeated transcranial magnetic stimulation (LF-rTMS) and superparamagnetic iron oxide nanoparticles (SPIONs) on repetitive digging behavior. The graph displays the results of the marble burying test, which assesses repetitive digging behavior. (N = 8, mean ± SEM). **p < 0.01, ***p < 0.001.

The study's findings indicated significant differences in the marble burying test (F (5, 42) = 5.482, *p* < 0.001; Fig. 3). Multiple comparisons revealed that autistic rats buried significantly more marbles compared to the sham group (*p* < 0.01). However, the LF + ASD group showed significant differences compared to the autistic group (*p* < 0.01). Additionally, treatment with SPIONs led to significant improvements in the LF + NP + ASD group (*p* < 0.001).

### Open field test

**Figure 4:**
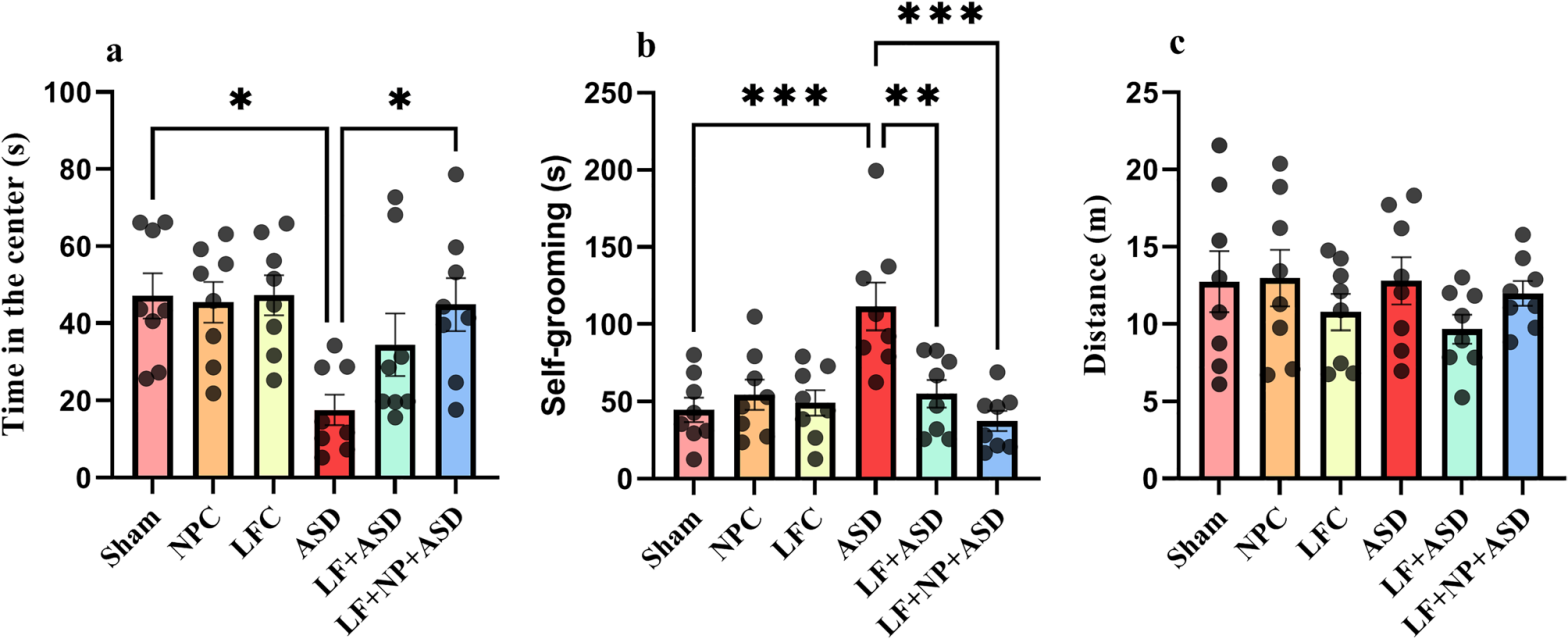
The effect of low-frequency repeated transcranial magnetic stimulation (LF-rTMS) and superparamagnetic iron oxide nanoparticles (SPIONs) on the open field test. (**a**) The time spent in the center of the apparatus as an indicator of anxiety behavior. (**b**) Self-grooming behavior representing repetitive and convulsive behavior. (**c**) The distance traveled through the apparatus during the experiment. (N = 8, mean ± SEM). *p < 0.05, **p < 0.01, ***p < 0.001.

The experiment aimed to measure anxiety-like behavior and locomotion in rats using an apparatus where spending more time in the center indicated less anxiety. One-way ANOVA showed significant differences in anxiety-like behavior between the groups (F (5, 42) = 3.805, *p* = 0.006; Fig. 4a). The data revealed that autistic rats spent significantly less time in the center of the apparatus compared to the sham group (*p* < 0.05). However, compared to the autistic group, the LF + NP + ASD group demonstrated reduced anxiety-like behavior (*p* < 0.05).

Self-grooming time was also recorded when the rats were in the open field. Significant differences were observed between groups (F (5, 42) = 7.314, *p* < 0.001; Fig. 4b). ASD rats exhibited significantly more self-grooming behavior compared to the sham group (*p* < 0.001). LF-rTMS significantly reduced this behavior (*p* < 0.01), and when this treatment was combined with SPION injection, self-grooming behavior decreased significantly even further (*p* < 0.001).

Additionally, the rat's locomotion behavior was recorded as the distance traveled in the apparatus. However, none of the groups showed significant differences in locomotion compared to the sham and ASD group (Fig. 4c).

### Forced swim test (FST)

**Figure 5:**
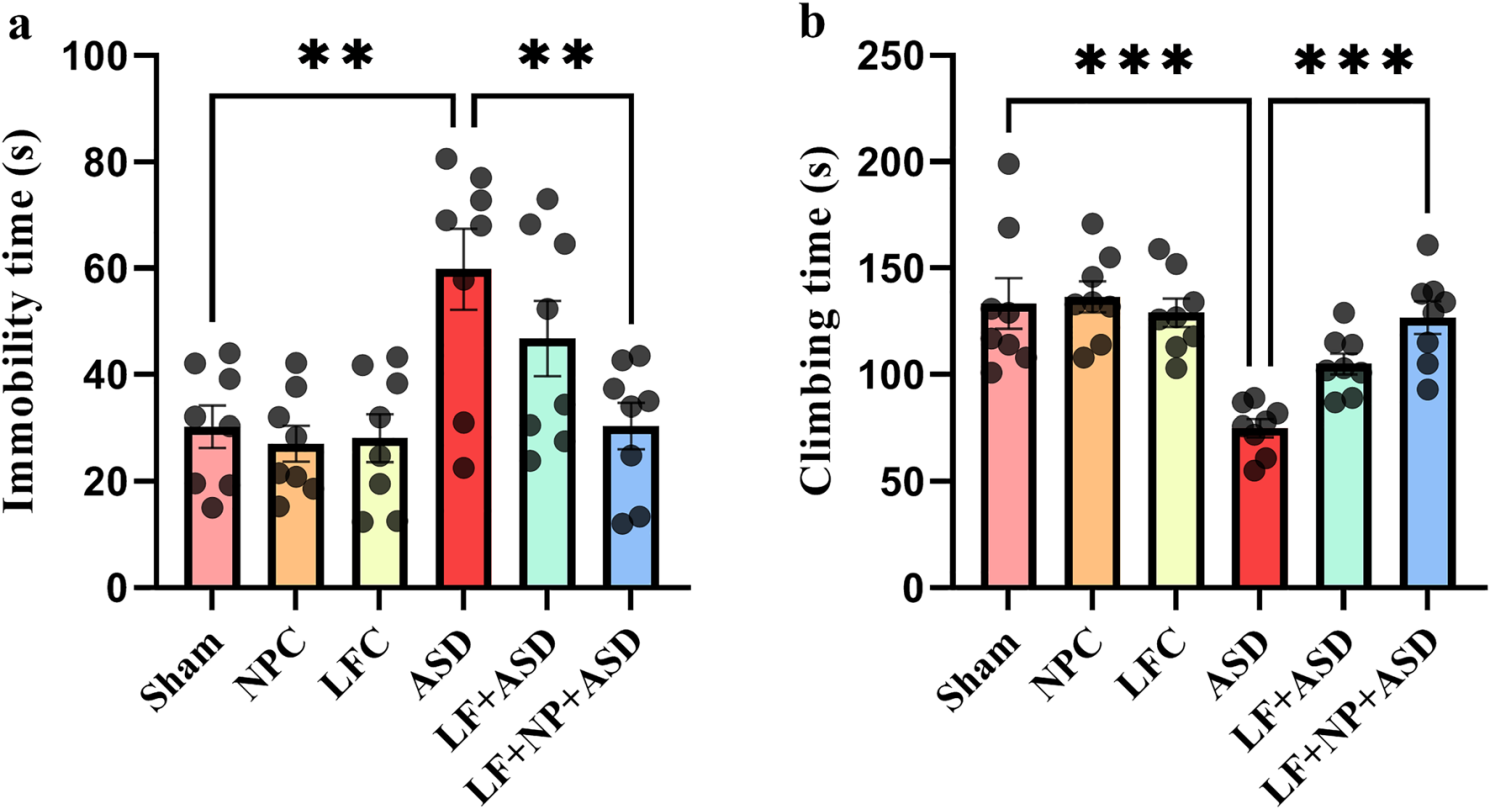
The effect of low-frequency repeated transcranial magnetic stimulation (LF-rTMS) and superparamagnetic iron oxide nanoparticles (SPIONs) on stress coping strategy. (**a**) Immobility time. (**b**) Climbing time. Both serving as markers for stress coping strategy. (N = 8, mean ± SEM). **p < 0.01, ***p < 0.001.

The results of the present study indicated a statistically significant difference in the FST among the groups regarding the time of immobility (F (5, 42) = 6.085, *p* < 0.001; Fig. 5a). Post hoc analysis revealed that the time of immobility in the ASD group was significantly higher compared to the sham group (*p* < 0.01). However, treatment with LF-rTMS with SPIONs significantly decreased the time of immobility in rats (*p* < 0.01).

Furthermore, concerning climbing time, there were also significant changes among the experimental groups (F (5, 42) = 9.877, *p* < 0.001; Fig. 5b). The autistic group exhibited a significant reduction in climbing time compared to the sham group (*p* < 0.001). Treatment in LF + NP + ASD groups showed significant increase in climbing time (*p* < 0.001).

### Biochemical parameters

**Figure 6:**
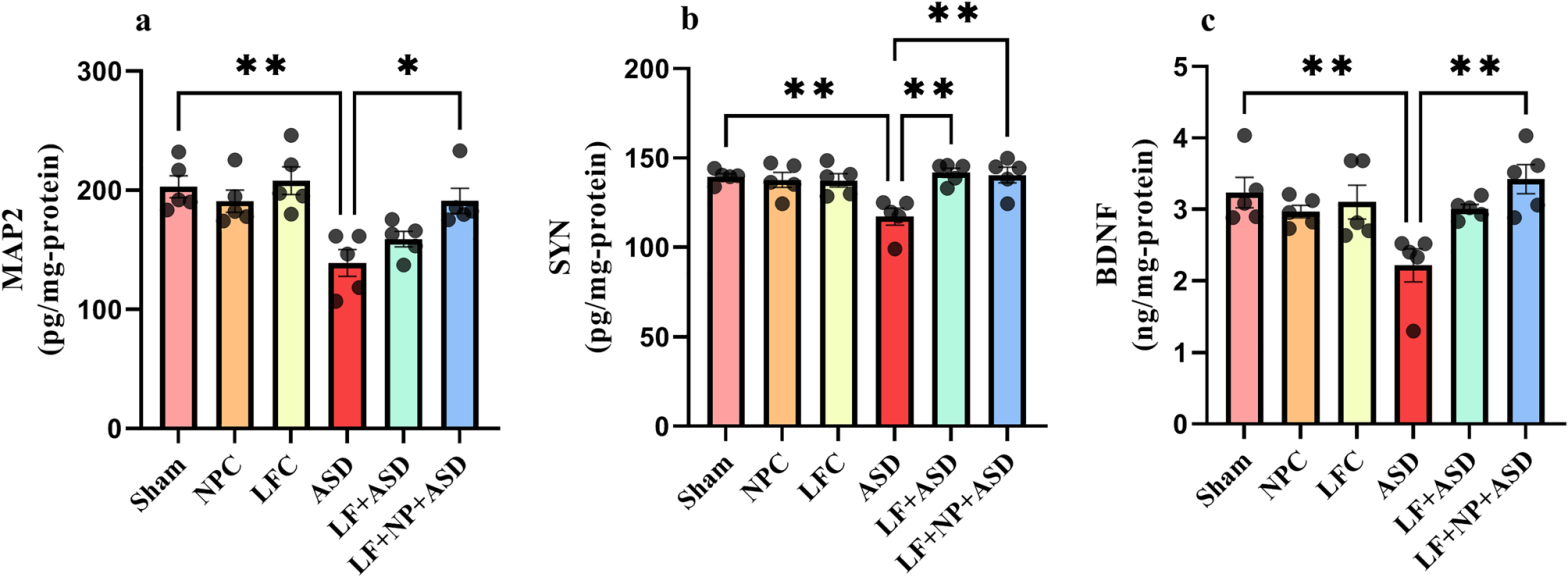
The effect of low-frequency repeated transcranial magnetic stimulation (LF-rTMS) and superparamagnetic iron oxide nanoparticles (SPIONs) on biochemical parameters of the hippocampus. (**a**) Microtubule-associated protein 2 (MAP2). (**b**) Synaptophysin (SYN). (**c**) Brain-derived neurotrophic factor (BDNF). (N = 5, mean ± SEM). *p < 0.05, **p < 0.01.

Statistical analysis of MAP2 levels in the hippocampus showed significant differences between groups in our study (F (5, 24) = 7.479, *p* < 0.001; Fig. 6a). The data demonstrated that the ASD group had significantly lower levels of MAP2 in hippocampal tissue compared to the sham group (*p* < 0.01). However, autistic rats treated with LF-rTMS with SPIONs showed a significant increase in MAP2 levels in the hippocampus (*p* < 0.05).

Furthermore, one-way ANOVA of SYN levels in the hippocampus also showed significant differences among groups (F (5, 24) = 6.175, *p* < 0.001, Fig. 6b). SYN levels in the hippocampus were reduced in the ASD group (*p* < 0.01), and treatment with LF-rTMS, with or without SPIONs, significantly increased SYN levels (*p* < 0.01).

Additionally, BDNF levels exhibited significant differences in this study (F (5, 24) = 4.946, *p* = 0.003, Fig. 6c). VPA exposure in rats significantly reduced BDNF levels compared to the sham group (*p* < 0.01). When this treatment was combined with SPION injection, BDNF levels increased significantly in this region (*p* < 0.01).

### Dendritic spine density

**Figure 7:**
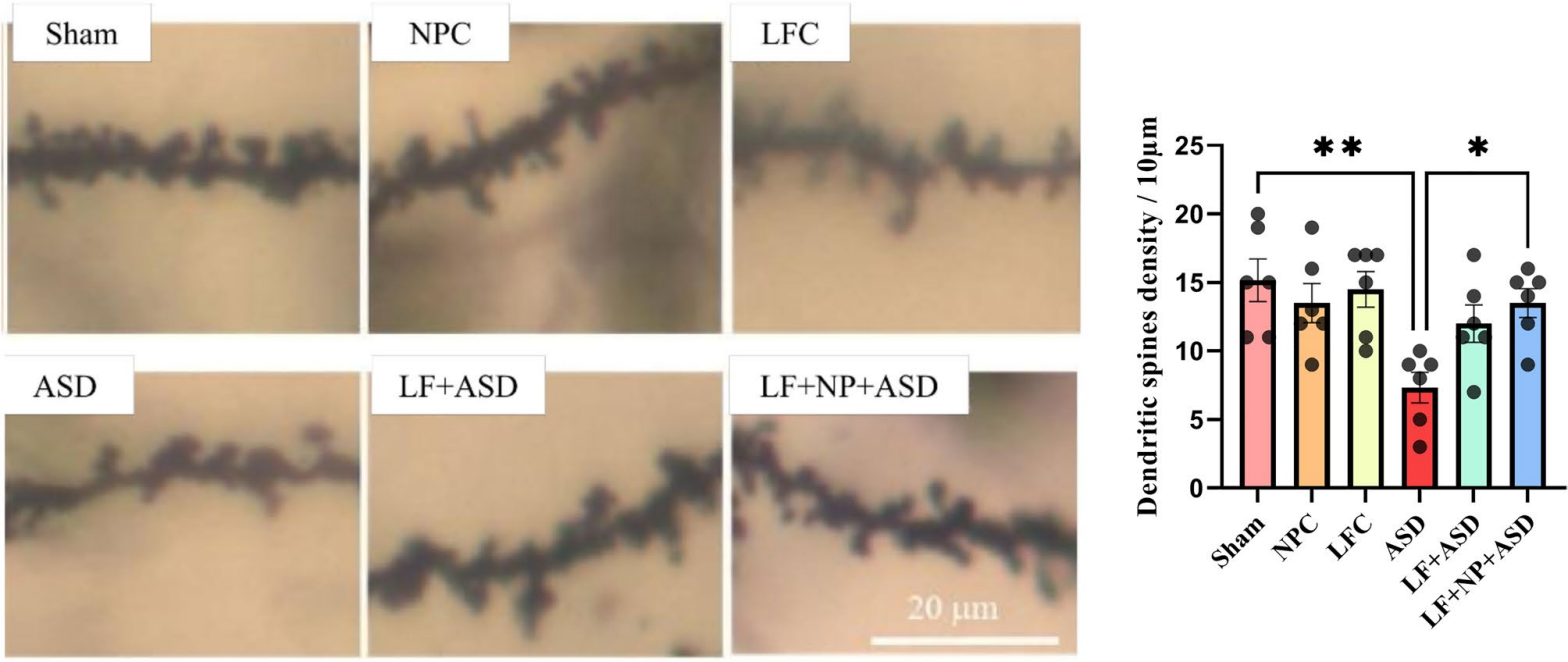
The effect of low-frequency repeated transcranial magnetic stimulation (LF-rTMS) and superparamagnetic iron oxide nanoparticles (SPIONs) on dendritic spine density. This graph displays images of dendritic spines in the CA1 region of the hippocampus and the density of spines in different groups. (N = 6, mean ± SEM). *p < 0.05, **p < 0.01.

Figure 7 displays the spine density in the CA1 area of the hippocampus. Spine density exhibited significant differences in this area between groups (F (5, 30) = 4.593, p = 0.003). The ASD group displayed significant reduced density compared to the sham group (p < 0.01), and LF-rTMS treatment with SPIONs significantly increased spine density in this area (p < 0.05).

## Discussion

In this study, our aim was to use LF-rTMS and SPIONs for the treatment of VPA-induced ASD model and investigate the potential mechanisms in the hippocampus. This is in consideration of the fact that the hippocampus plays a significant role in the pathophysiology of the VPA model of ASD. For this purpose, we demonstrated the dysfunctions caused by prenatal VPA in this model at the behavioral, biochemical, and histological levels. At the behavioral level, our data showed major dysfunction in social behavior, anxiety and repetitive behavior. Furthermore, we observed a reduction in stress coping strategies in this model, which is noteworthy because the hippocampus plays a key role in stress management^30^. These behavioral dysfunctions are associated with reduced MAP2, SYN, and BDNF levels in the hippocampus, as well as a decrease in CA1 hippocampal dendritic spine density. After treatment with LF-rTMS, we observed some improvement. However, when this treatment was combined with SPION injection, the effectiveness of the treatment increased.

Previous studies suggest that rTMS can affect brain excitability, behavior, and physiological changes. Repeating rTMS for several sessions has cumulative effects, and all the mentioned outcomes become more significant. Magnetic pulses generated with a coil induce an electrical field when they reach the neurons, causing neural stimulation. The final outcome may be seen in a change in synaptic efficacy related to the excitability of the neuron and other chemical factors like BDNF^31^. This may eventually lead to changes in the behavioral symptoms of ASD.

Studies suggest that LF-rTMS may lower the risk of seizures in patients, primarily due to its inhibitory effects on the cortex when compared to HF-rTMS^18,32^. Moreover, multiple studies have explored the potential therapeutic benefits of LF-rTMS for both individuals with autism and animal models^18,33,34^.

While there have been reports suggesting that a human coil can stimulate the entire brain of small rodents^35^, it is worth noting that evidence indicates a reduction in magnetic field intensity with increased distance from the surface of the coil^36^. To address this limitation, some research has shifted focus toward the utilization of SPIONs and their magnetoelectric properties to effectively stimulate specific brain regions^37^. SPIONs, when exposed to a magnetic field such as that produced by TMS, can generate local magnetic pulses and stimulate surrounding areas^29,37^. These particles exhibit remarkable stability both outside and inside the body.

Our findings demonstrated that chitosan-coated SPIONs remain in the hippocampus for a period of one month. This aligns with a prior study in which Fe_3_O_4_ particles persisted in the brain for over a month^25^. Additionally, our TEM images and FTIR spectra indicate the presence of well-formed chitosan-coated SPIONs and the successful bonding of chitosan with Fe_3_O_4_. These findings are also consistent with previous research on these particles^38^.

Studies have reported that the consumption of VPA by pregnant women during early pregnancy may increase the risk of ASD in their children^39^. Consequently, many studies suggest using VPA to induce ASD in small rodents^3^. There are several protocols for ASD induction in rodents with VPA. One study aimed to compare prenatal VPA-induced ASD to a postnatal VPA model, and the results suggested that ASD symptoms are more pronounced in offspring in the prenatal model, with a much lower mortality rate in offspring^40^. Although the most commonly used model for ASD induction is 600 mg/kg VPA at 12.5 days of pregnancy, this period is crucial^41^. Studies have shown that day 12 of pregnancy in rats is the most critical day for ASD and social impairment induction^42^. Furthermore, several clinical and preclinical studies have explored the use of TMS as a therapeutic intervention for ASD^18,43,44^.

Our data showed that LF-rTMS intervention has the potential to reverse core autism behavioral deficits in the VPA model of ASD, similar to findings described in another study involving a maternal separation-induced ASD model^18,22^. Interestingly, when this treatment is combined with SPION injection, it appears to have a stronger impact on behavioral characteristics. This effect is in line with findings from another study involving a depression model^28^.

Atypical hippocampal development in ASD has recently received increased attention in research. This dysfunction is associated with deficits in memory processing, social interaction, and spatial reasoning^5^. Studies have suggested that the CA1 area of the hippocampus plays a crucial role in social memory, which is essential for social interactions in animals^45^. In our studies, we observed reduced dendritic spine density in the CA1 area of the hippocampus in autistic rats. Another study also demonstrated a reduction in dendritic spine density in this region when animals were exposed to prenatal VPA^46^.

Our results further indicate that treatment with LF-rTMS + SPIONs can increase spine density in this area. From another perspective, studies have reported that MAP2 is associated with spine density and structures^47^. Our findings from MAP2 also support the spine density results obtained through Golgi-Cox staining. Additionally, an important factor influencing dendritic spines in the CA1 area is BDNF. Research has shown that BDNF can impact dendritic spine density and dendritic length in this region^48^.

SYN is well known for its involvement in synaptic plasticity mechanisms, and it is one of the most crucial proteins within synaptic vesicles. The synaptic vesicle is a site within the presynaptic neuron where neurotransmitters are stored^11^. An increase in SYN levels can enhance neurotransmitter secretion into the synaptic space, thereby facilitating synaptic transmission between neurons^49^. Our results indicated a reduction in SYN levels in the hippocampus of autistic rats, and LF-rTMS therapy has been shown to increase SYN levels, consequently enhancing neural transmissions.

One limitation relates to the size of the rat brain, posing a challenge in focusing TMS coil stimulation specifically on the hippocampus. Unintended stimulation of other brain areas may influence our results. Using a smaller coil with a new design could potentially improve the precision of stimulation in the desired area. Additionally, the injection of SPIONs into the lateral ventricle may result in their dispersion with cerebrospinal fluid to other brain regions rather than concentrating in the hippocampus. For future studies, exploring alternative methods, such as direct injection of SPIONs into the hippocampus or developing less invasive ways to target SPION delivery to the hippocampus, would be beneficial. Another limitation of our study is related to the timing of behavioral analysis to assess the long-term effect of TMS. It may be worthwhile to conduct behavioral and molecular analyses several months after treatment. Furthermore, assessing gender diversity to identify sex-dependent effects of this treatment on the ASD model is worthwhile. Due to the wide-ranging effects of VPA on the body, future studies may benefit from specific pharmacological methods or genetic models. This is another limitation of our study.

Our study suggests that ASD involves dysfunction in hippocampal dendritic spines and neural transmission, which can also have an impact on several core ASD behaviors. LF-rTMS treatment, when combined with SPION injection, has the potential to improve behavioral symptoms associated with ASD. The increased spine density in the CA1 area of the hippocampus is associated with this treatment and is mediated by an increase in BDNF, MAP2, and SYN levels. Furthermore, our research highlights the success of deep brain stimulation with SPIONs, offering a promising avenue for further investigation into the effects of TMS on deep brain structures and the development of novel treatments for neurological disorders.

## Methods

### Animal study and experimental procedure

This study adhered to the ARRIVE guidelines. This study comply with the rules and guidelines of Shahid Beheshti University. The study was approved by Research Ethics Committees of Shahid Beheshti University with approval ID: IR.SBU.REC.1401.108. Adult Wistar rats were housed in standard animal facilities, maintaining controlled conditions (22 ± 1 °C, humidity: 45 ± 3%, and a 12-h light/dark cycle). The rats had free access to standard food and water. Male and female rats were co-housed in cages with nesting materials, and successful mating was confirmed by the presence of a white plug in the vagina or cage.

**Figure 8:**
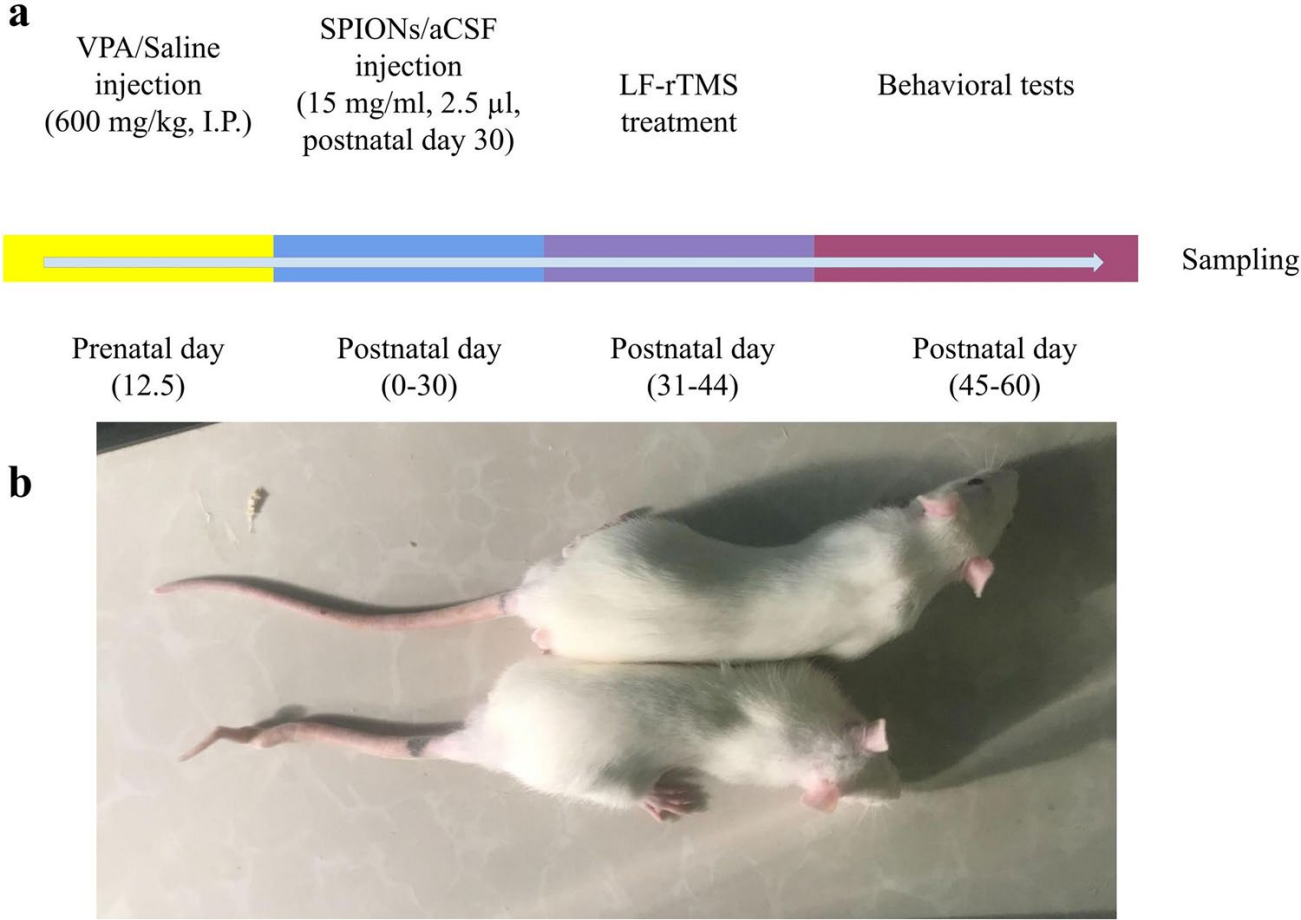
The timeline of the study and anatomical malformation caused by prenatal valproic acid (VPA). (**a**) After mating on the 12.5th day of pregnancy, valproic acid (VPA) or saline was injected intraperitoneally (600 mg/kg) to induce autism spectrum disorder (ASD). On postnatal day 30, superparamagnetic iron oxide nanoparticles (SPIONs) were injected into the lateral ventricle of the brain (15 mg/ml, 2.5 µl). From days 31 to 44, low-frequency repeated transcranial magnetic stimulation (LF-rTMS) treatment was administered for 14 consecutive days. Subsequently, rats were subjected to behavioral testing, and on day 60, rats were sacrificed for brain sample collection for further analysis. (**b**) Anatomical malformation of the tail of a rat compared to a healthy rat after prenatal VPA exposure.

Following successful mating (Fig. 8a), female rats were randomly divided into two groups: one group received vehicle, while the other group received an intraperitoneal injection of 600 mg/kg of VPA (Darou Pakhsh Co., Tehran, Iran) at 12.5 days after successful mating^50^. One of the side effects of VPA on 100% of rat offspring is a twisted tail observed at one or several locations, as shown in Fig. 8b. Later, on postnatal day 21, male rats were separated from the others and randomly assigned to one of six groups, each consisting of 8 rats: Sham, Control + SPION (NPC), Control + LF-rTMS (LFC), VPA (ASD), ASD + LF-rTMS (LF + ASD), and ASD + SPION + LF-rTMS (LF + NP + ASD).

On day 30, stereotaxic surgery was performed under ketamine/xylazine anesthesia to inject SPION solution (15 mg/ml, 2.5 µl) into the lateral ventricle of the brain.

### Repetitive transcranial magnetic stimulation (rTMS)

According to a previous study, rats underwent LF-rTMS (1 Hz, 20 trains, 30 pulses in each train, with 2-s inter-train intervals, totaling 600 pulses)^18^ from postnatal day 31 to 44, conducted between 8 a.m. and 12 p.m. Throughout the experiments, the stimulation intensity for LF-rTMS sessions remained consistently set at 100% of the average resting motor threshold (50% of the maximum output). The determination of this threshold was established through an initial experiment conducted on conscious animals. In this experiment, the average stimulation intensity required for bilateral forelimb movement was evaluated through visual observation in a group of 4–5 rats^18^.

To ensure the rats immobility during the experiment, a cloth restraint based on a previous study^51^ was utilized. The TMS stimulator employed in this research was the Super Rapid 2 device (MagStim, UK), equipped with a 70 mm human 8-shaped coil (D70 air film coil). To acclimate the rats and minimize stress, they were restrained and exposed to TMS device noise for one week prior to the experiment. All treatments were administered in a quiet, low-light environment to minimize potential stressors. During the experiment, the coil was positioned over the area between the eyes and ears of the rats. For the sham group, the coil was oriented in the opposite direction to ensure that no electromagnetic stimulation was delivered, while the auditory conditions remained unchanged.

### SPIONs coating

To prepare coated nanoparticles (Fig. 1g), a mixture was created by combining 11.60 ml of acetic acid (MERCK, Germany) with 40 mg of chitosan (Iran Chitosan, Iran), and 0.14 g of iron-oxide nanoparticles (Fe_3_O_4_) (Arminano, Iran) was added to 200 ml of distilled water and stirred for 15–20 h. This resulted in a color change from black to brown. The mixture was then subjected to centrifugation and washed twice with distilled water. Finally, the nanoparticles were dried at 65 °C to obtain a powdered form^52^.

The morphology of the coated SPIONs was examined using a transmission electron microscope (TEM, Philips EM 208). The hydrodynamic diameter/zeta potential of the nanoparticles were measured using a HORIBA S-Z100 instrument. Fourier transform infrared spectroscopy (FTIR) was employed to identify the chemical groups and interactions in the chitosan-coated SPIONs (Bomem, Japan). Additionally, the magnetic hysteresis curve was measured using a vibrating sample magnetometer (VSM) from Magnetic Kavir Kashan Co., Iran.

### Assessment of cytotoxicity

Following the previously described method^53^, the MTT assay was employed to assess the cytotoxic effects of SPION on human neuroblastoma cells (SH-SY5Y). Various SPION concentrations (ranging from 0 to 100 μg/ml) were introduced to the cells in 96-well plates and incubated for 24 h. Cell viability was subsequently determined using a microplate reader at 570 nm and compared to the untreated control group.

### Three chamber test

To assess social behavior and the ability to adapt to new social stimuli, the researchers employed the three-chamber task (Fig. 2d). The experimental setup consisted of an 80 × 80 × 40 cm Plexiglass box divided into three equal chambers. The task involved three stages: familiarization, social interaction, and novelty response^2^.

In the first stage, the rat was introduced into the center chamber of the empty apparatus and allowed to explore for a duration of 10 min. Subsequently, in the second stage (10 min), one of the side chambers housed an unfamiliar rat, while the other remained unoccupied but contained a metal cage. The test rat was then returned to the center chamber to assess its level of social interaction and sniffing behavior.

In the third stage (10 min), the metal cage in one of the chambers was filled with a new, unfamiliar rat, while the metal cage in the other chamber still contained the same rat (familiar rat) from the previous stage. This step aimed to evaluate social novelty.

### Marble burying test

The marble burying test was conducted in a 40 × 40 × 40 cm box containing 20 glass marbles arranged in 5 rows. The test spanned 30 min, during which the rats were permitted to interact with the marbles. Upon completion of the test, the researchers counted the number of marbles that had been buried under the bedding material as an indicator of repetitive digging behavior^2^.

### Open field test

In the open-field test, each rat was positioned in a 40 × 40 × 40 cm box that was partitioned into a central circular area and squares surrounding it. The rat was allotted 15 min to explore the box, while various parameters were observed. These parameters included the time the rat spent in the central circular area, the duration of self-grooming behavior, and the distance traveled by the rat^2^.

### Forced swim test

Rats were subjected to a swimming test in a cylindrical plastic container filled with water (30 cm in diameter and 50 cm in height) maintained at a temperature of 25 ± 1 °C^54^. The rats were given two minutes to acclimate to the cylinder. This test measures the response to acute stress, which may be influenced by certain neurological disorders, such as ASD^55^. The duration of immobility and climbing time was subsequently monitored for 5 min. Following the completion of each swimming test, the rats were gently dried with a towel, and the water in the tank was replaced.

### Biochemical parameters

At post-natal day 60, following the behavioral tests, rats were anesthetized with a combination of ketamine/xylazine (80 mg/kg and 10 mg/kg). Subsequently, the brains were removed, and the hippocampus was homogenized with a lysis buffer solution for further processing.

### Microtubule-associated protein 2 (MAP2)

The samples were assessed using a conventional MAP2 sandwich ELISA procedure, as detailed in a prior research publication^56^.

### Synaptophysin (SYN)

The ELISA procedure for quantifying synaptophysin concentration was conducted following the methodology outlined in a previously referenced study^57^ with an ELISA kit (CSB-E13827r). Briefly, the synaptophysin ELISA involves preparing samples, coating the microtiter plate with synaptophysin antibody, adding samples or standards, incubation, washing, adding detection antibody conjugated to an enzyme, incubation, washing again, adding TMB Development Solution for color development, adding Stop Solution to halt the reaction, and measuring signal intensity at 450 nm for quantitative analysis of synaptophysin levels.

### Brain-derived neurotrophic factor (BDNF)

The assessment of supernatant BDNF levels was conducted by employing a BDNF ELISA kit (RAB 1138, Sigma, USA), adhering strictly to the manufacturer's guidelines. The rate of the reaction was determined using a microplate reader (Bio Tek, USA) set to a wavelength of 450 nm.

### Protein assessment

To quantify the protein content in the samples, we utilized the BCA (bicinchoninic acid) method. The absorbance at 562 nm was measured to quantify the amount of protein in accordance with a previous study^58^.

### SPIONs content measurement

In accordance with a previously published article^59^, the SPIONs content in the hippocampus was measured in the supernatant. Briefly, to develop color in a 96-well plate, hippocampus supernatant was mixed with an iron assay solution, and the absorbance was read at 562 nm. The data were expressed as µg/ml.

### Golgi-Cox staining

The Golgi impregnation method was employed as described previously^60^. Brain blocks were fixed and immersed in a solution for two weeks (1% mercury chloride, 0.8% potassium chromate, 1% potassium dichromate, 0.5% potassium tungstate), followed by transfer to another solution (1% lithium hydroxide and 15% potassium nitrate). Subsequently, the brains were placed in a sucrose-buffer solution, and 100 μm slices were prepared using a cryotome. Spine density in the CA1 hippocampal area was calculated using ImageJ software.

### Statistical analysis

For data analysis, we utilized GraphPad Prism (ver. 9.5.1). The statistical analysis comprised a one-way ANOVA, followed by a Tukey test. The Shapiro–Wilk test was employed to assess normality, and ROUT (Q = 0.5%) analysis was used to identify outliers. The homogeneity of variance was calculated using the Brown-Forsythe test. The results are presented as the mean ± SEM, and *p* values less than 0.05 were considered statistically significant.

## Data Availability

The datasets generated during and/or analyzed during the current study are available from the corresponding author on reasonable request.
